# Mucoid degeneration of the posterior cruciate ligament in a college soccer player: a case report

**DOI:** 10.1186/s13256-021-02893-4

**Published:** 2021-06-03

**Authors:** Ryo Kanto, Hiroshi Nakayama, Tomoya Iseki, Shintaro Onishi, Ryo Iwakura, Shinichi Yoshiya, Toshiya Tachibana

**Affiliations:** 1 Department of Orthopaedic Surgery, Hyogo College of Medicine, 1-1 Mukogawa-cho, Nishinomiya, Hyogo 663-8501 Japan; 2 Department of Orthopaedic Surgery, Nishinomiya Kaisei Hospital, Nishinomiya, Japan

**Keywords:** Mucoid degeneration, PCL, Young athlete

## Abstract

**Background:**

To the best of our knowledge, arthroscopic treatment for symptomatic mucoid degeneration of the posterior cruciate ligament in young athletes has not been reported before.

**Case presentation:**

An 18-year-old Asian male college soccer player presented with a 3-month history of right knee pain without episodes of trauma. Despite conservative treatment over the preceding 3 months, his symptoms persisted. Physical examination of the right knee revealed full range of motion, though posterior knee pain was induced when the knee approached full flexion. On ligament examination, posterior sagging and Lachman test were negative, and no clinical finding indicative of ligament insufficiency was noted. Magnetic resonance imaging showed a diffusely thickened posterior cruciate ligament with increased signal intensity on the T2-weighted sequence. A few intact fibers were observed with continuous margin from origin to insertion. Based on the patient's history and the magnetic resonance imaging findings, we suspected mucoid degeneration of the posterior cruciate ligament as the cause of the patient’s symptoms. Since conservative treatment had failed to relieve the symptoms, arthroscopic treatment was indicated. Arthroscopic examination revealed yellowish crumbly tissues along the thickened posterior cruciate ligament. Tension and bulk of the posterior cruciate ligament were well preserved. Curettage of degenerative tissue and decompression of the posterior cruciate ligament resulted in symptom relief without instability of the knee joint. The patient returned to play at 3 months. At 12 months, postoperative magnetic resonance imaging showed no evidence of recurrence and indicated that the remaining posterior cruciate ligament was thicker than before the surgery. At 2 years follow-up, the patient remained asymptomatic and could play soccer at the same level as before the onset of pain.

**Conclusions:**

Arthroscopic decompression of the posterior cruciate ligament may relieve knee pain and facilitate early return to play with good functional results.

## Background

Although mucoid degeneration of anterior cruciate ligament (ACL) has been sporadically reported [1], the same pathology in the posterior cruciate ligament (PCL) is less common, with only a small number of cases reported in English literature [2–6]. To our knowledge, arthroscopic treatment for symptomatic mucoid degeneration of the PCL in athletes has not been reported before. We report herein a case of mucoid degeneration of the PCL with complaints of posterior knee pain during sports activity, which was successfully managed with arthroscopic debridement.

## Case presentation

**Fig. 1:**
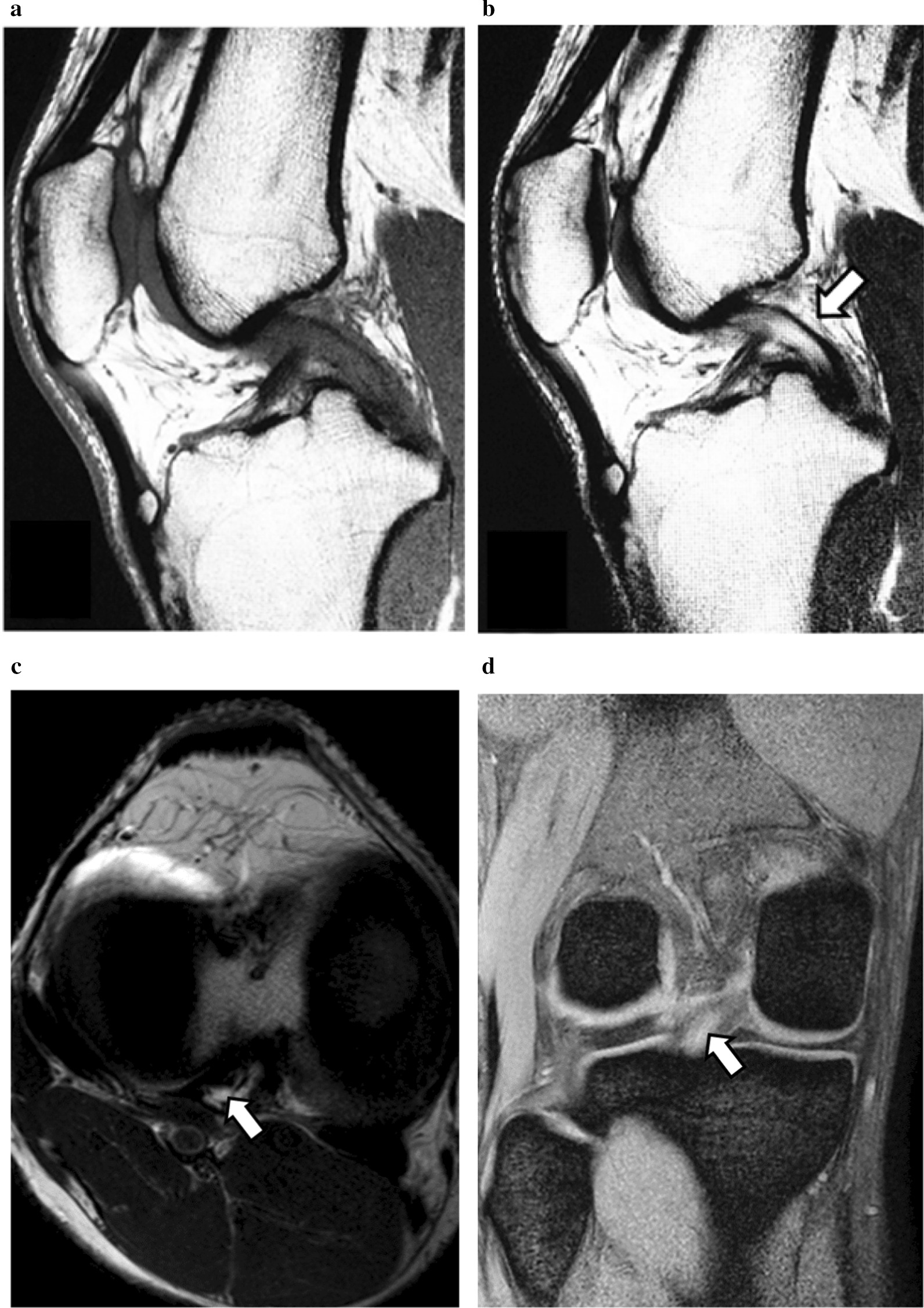
Preoperative MRI. **a** Sagittal T1-weighted image of the PCL showing relative increased signal within ligament consistent with diffuse mucoid degeneration. **b** Longitudinal increased signal intensity area within the PCL on sagittal T2-weighed images with an adjacent well-defined rim of hypointense PCL fibers (white arrow). **c** Axial and **d** coronal T2-weighed images of the PCL showing high signal intensity area within the PCL (white arrow)

An 18-year-old Asian male college soccer player presented with a 3-month history of right knee pain. Prior to the onset of symptoms, no episodes of trauma or previous knee problems were noted. Despite conservative treatment over the preceding 3 months consisting of stretching, isometric quadriceps/hamstring strengthening exercises, and medication, his symptoms persisted, and he had not been able to participate in practice sessions or games. The pain aggravated with increased activity. Physical examination of the right knee revealed full range of motion, though posterior knee pain was induced when the knee approached full flexion. On ligament examination, posterior sagging and Lachman test were negative, and no clinical finding indicative of ligament insufficiency was noted. Radiograph of the knee was normal. Magnetic resonance imaging (MRI) revealed longitudinal increased signal intensity area within the PCL on T2-weighed images with an adjacent defined rim of hypointense PCL fibers (Fig. 1). The characteristics of those images were coincident with a “Tram-Track” appearance according to the description by McMonagle *et al.* [7]. Based on the patient’s history and the MRI findings, we suspected mucoid degeneration of the PCL as the cause of the patient’s symptoms. Since conservative treatment had failed to relieve the symptoms, arthroscopic treatment was indicated.

**Fig. 2:**
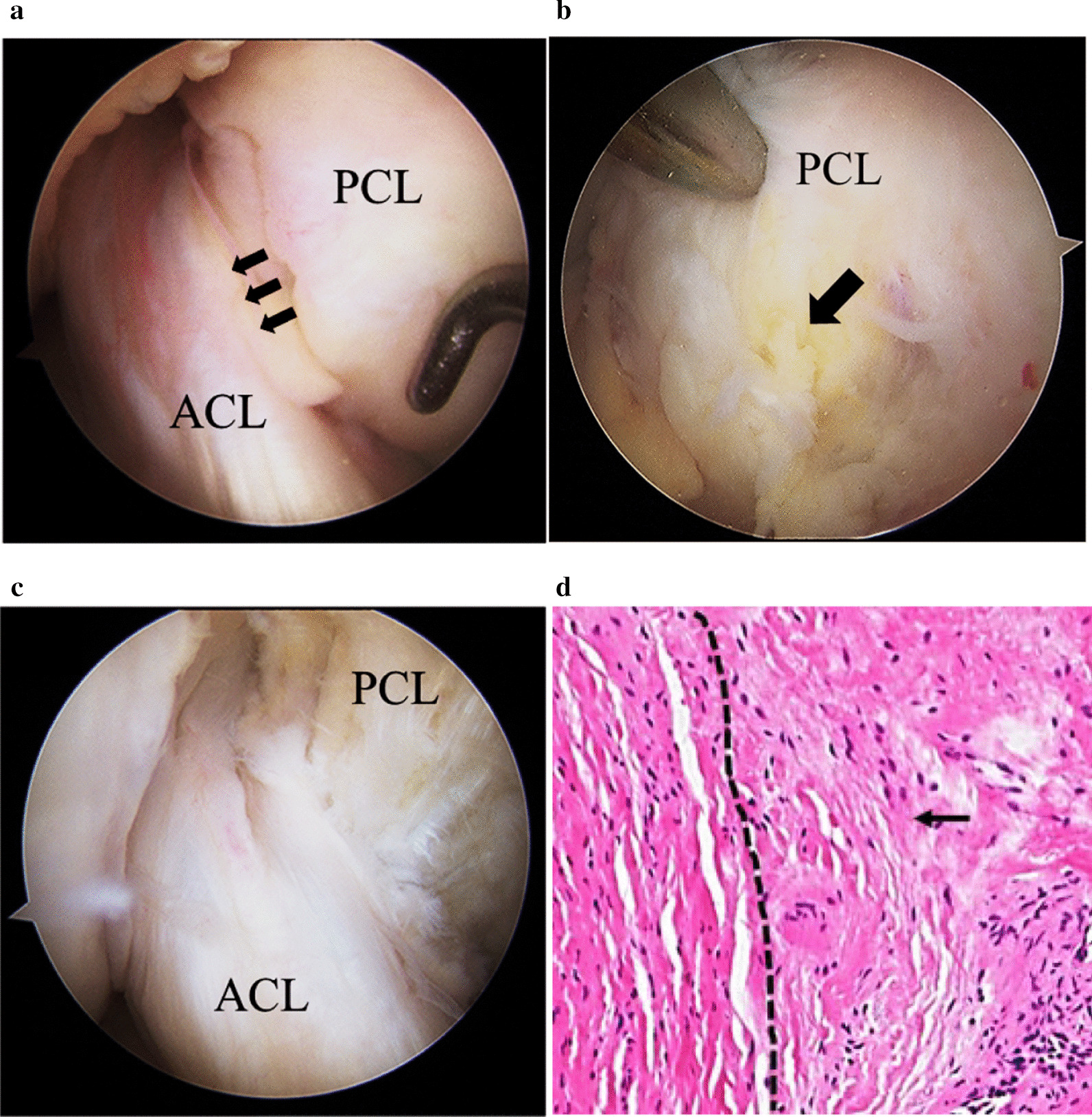
Arthroscopic findings of the knee and histological examination of PCL. **a** Arthroscopic examination from anterior view revealed yellowish crumbly tissues along the thickened PCL. Tension and bulk of the PCL were well preserved. Anterior cruciate ligament appearance and tension with probing was normal but was pushed towards lateral wall of the notch (black arrow). **b** The yellowish tissue was present in deeper region (black arrow) while retention of fluid was not observed within the ligament substance. **c** The PCL tissue was split, and evidently degenerated tissue was carefully removed while meticulous care was taken to preserve the longitudinal fiber of the PCL. MFC; medial femoral condyle. **d** On histologic examination of the PCL, loose fibrocollagenous tissue containing myxedematous changes indicated mucoid degeneration of the ligament (right side of a dotted line, black arrow, Hematoxylin and eosin stain)

Arthroscopic examination revealed yellowish crumbly tissues along the thickened PCL. Tension and bulk of the PCL were well preserved. Other intraarticular tissues such as ACL, menisci, and cartilage were intact. The PCL tissue was split, and evidently degenerated tissue was carefully removed while meticulous care was taken to preserve the longitudinal fiber of the PCL. On arthroscopic observation, the yellowish tissue was present in deeper regions while retention of fluid was not observed within the ligament substance. Histological examination of the specimen obtained from the yellowish degenerative lesion of PCL revealed loose fibrocollagenous tissue containing myxedematous changes indicative of mucoid degeneration of the ligament (Fig. 2).

**Fig. 3:**
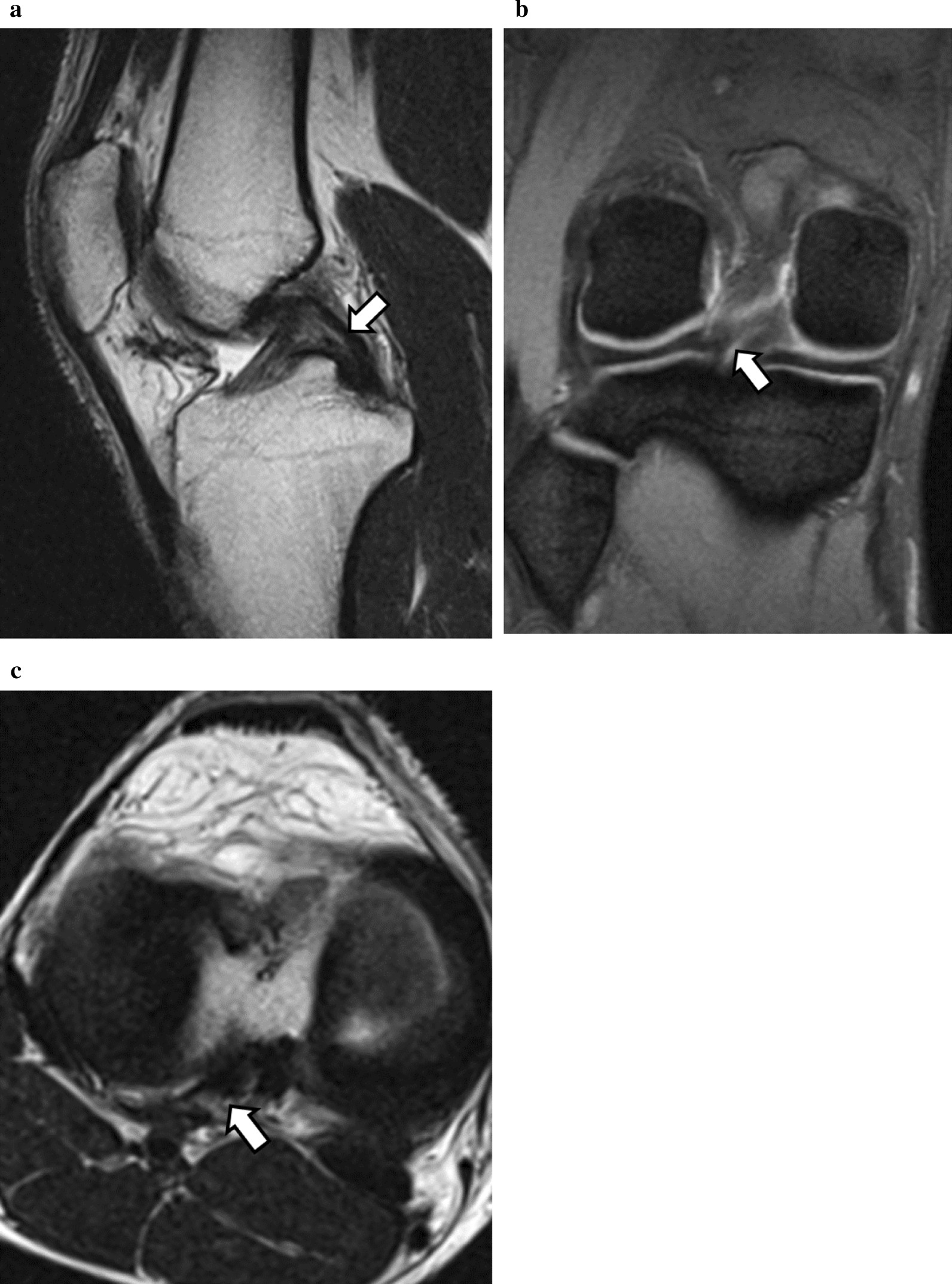
Postoperative MRI at 12 months after surgery. At 12 months postoperative sagittal (**a**), coronal (**b**), and axial (**c**) T2-weighted MRIs showed no evidence of recurrence and indicated that the remaining PCL was thicker than before the surgery (white arrow)

Postoperatively, the patient was allowed to bear full weight on the following day. Rehabilitation protocol including early motion and quadriceps strengthening exercise was started from the first postoperative week. Running and agility training was started at 6 weeks. The patient returned to play at 3 months. At 12 months, postoperative MRIs showed no evidence of recurrence and indicated that the remaining PCL was thicker than before the surgery (Fig. 3). At 2 years follow-up, the patient remained asymptomatic and could play soccer at the same level as before the onset of pain. The preoperative International Knee Documentation Committee knee subjective score of 40.2 points improved to 98.6 points at 2 years after surgery. The patients and their families were informed that data from the case would be submitted for publication, and gave their consent.

## Discussion

Although rare, mucoid degeneration of the PCL can be a source of activity-related pain in athletes. As a point of reference, Bergin *et al.* [1] reported that the incidence of mucoid degeneration in ACL is 1.0% (44/4221), and McMonagle *et al.* [7] reported that the incidence of PCL mucoid degeneration is 0.1% (14/12,972). To our knowledge, only five cases of symptomatic mucoid degeneration of the PCL have been previously reported. A review of these five cases indicated that mucoid degeneration of the PCL generally affects middle-aged patients with ages ranging from 36 to 65 years, in contrast to the present patient, who was 18 [2–6]. Mucoid degeneration of the cruciate ligaments is usually asymptomatic, and is often hard to diagnose clinically owing to the lack of specific symptoms and signs. However, MRI findings in sagittal plane show longitudinal layers of increased signal intensity on T2-weighted images within the ligament substance with an adjacent well-defined intact rim of hypointense PCL fibers that give the PCL a “tram-track” appearance [7]. Its clinical symptoms were characterized by posterior knee pain on deep knee flexion without causative or preceding injuries. To date, the mechanism underlying knee pain in mucoid degeneration of the PCL is controversial. According to previous studies, increased volume of the PCL and resultant increases in internal pressure within the notch have been considered a cause of knee pain during terminal flexion [6]. Several possible etiologies have been suggested, including structural variations of the intercondylar notch and microtrauma leading to the release of a mucin substance [8]. In this case, there was no abnormality in the morphology of the intercondylar notch. Therefore, we considered that excessive stress on the knee associated with repetitive minor injuries during the sports activity may have caused mucoid degeneration. For treatment of mucoid degeneration of the cruciate ligament, partial excision or debridement is usually performed. Most authors have reported satisfactory results after these procedures without instability [2–6]. In our case, we excised the PCL in part, and it resulted in complete relief of pain and full range of motion without instability. Mucoid degeneration of the PCL can be a source of activity-related pain in young athletes. Its clinical symptoms were characterized by posterior knee pain on deep knee flexion without causative or preceding injuries.

## Conclusion

We conclude that arthroscopic decompression of the PCL may relieve knee pain and facilitate early return to play with good functional results.

## Data Availability

The dataset used and analyzed during the current study is available from the corresponding author on reasonable request.

## Abbreviations

PCL: Posterior cruciate ligament
ACL: Anterior cruciate ligament
MRI: Magnetic resonance imaging

## Declarations

### Acceptable secondary publication

This article is based on a study first reported in the Seikeigeka 2019 Jan;70(1): 26–29. (in Japanese) [9]. The authors have received approval of secondary publication from the editors of “Seikeigeka” (in Japanese).
